# Audit of Nicotine Replacement Therapy Practices and Vaping Use in Inpatients: Adherence to Dual NRT Regimen and Documentation of Smoking Cessation Plans

**DOI:** 10.1192/bjo.2025.10557

**Published:** 2025-06-20

**Authors:** Marianne Bergman, Akeem Sule

**Affiliations:** Essex Partnership Foundation Trust, Wickford, United Kingdom

## Abstract

**Aims:** This audit aimed to assess nicotine use patterns in inpatients and evaluate adherence to the Essex Partnership University Trust Nicotine prescribing guidelines, as well as British Thoracic Society’s recommended dual nicotine replacement therapy (NRT) regimen. It also aimed to examine the documentation of smoking status, NRT usage, and cessation planning.

**Methods:** A sample of 40 inpatients was selected from 4 Mid-Essex general adult inpatient wards, using a random number generator. Data was collected from PARIS inpatient admission assessments, physical health checks (PHCs) and paper or online prescription charts to identify smoking status, nicotine use, and cessation discussions. Nursing staff provided information on vaping. The audit assessed whether patients were on dual NRT (short-acting and long-acting forms) and whether cessation plans were documented for patients using inhalators or vapes.

**Results:** The audit revealed that 57% of patients were smokers, and 72% of smokers were using vapes. None of the patients were receiving dual NRT, and no cessation plans were found for those using any form of NRT. While 75% of patients had a documented discussion about smoking and nicotine use, not all included NRT options. Vaping use was poorly documented, and most patients relied solely on short-acting NRT, such as vapes and inhalators. This lack of adherence to best practice creates challenges, especially during busy on-call shifts when short acting NRT prescriptions are frequently requested. Additionally, smoking cessation discussions were not consistently revisited, and vaping use was poorly documented. The absence of structured cessation strategies, including plans for maintenance or weaning, indicates a need for clearer management of nicotine dependence.

**Conclusion:** This audit recommends that every patient be asked about smoking status and that electronic records be updated accordingly. This should be re-visited if not possible initially. A dual NRT regimen (nicotine patch plus a fast-acting NRT) should be initiated as soon as possible. Vaping can be used as a short-acting method but not concurrently with other short-acting options, and needs to be clearly documented. Smoking cessation discussions should be consistently documented in PHCs, and all patients starting NRT should have a documented management plan. Furthermore dual NRT therapy should be incorporated into a prescribing bundle. We recommend a follow-up audit in 4–5 months to assess improvements in adherence to dual NRT therapy and vaping reliance.

